# Night Shift Work, DNA Methylation and Telomere Length: An Investigation on Hospital Female Nurses

**DOI:** 10.3390/ijerph16132292

**Published:** 2019-06-28

**Authors:** Michele Carugno, Cristina Maggioni, Eleonora Crespi, Matteo Bonzini, Simone Cuocina, Laura Dioni, Letizia Tarantini, Dario Consonni, Luca Ferrari, Angela Cecilia Pesatori

**Affiliations:** 1 Department of Clinical Sciences and Community Health, Università degli Studi di Milano, Via San Barnaba 8 – IT-20122 Milan, Italy; 2 Occupational Health Unit, Fondazione IRCCS Ca’ Granda Ospedale Maggiore Policlinico, Via San Barnaba 8 – IT-20122 Milan, Italy; 3 Occupational Health Unit, ASST Santi Paolo e Carlo, Via Antonio di Rudinì, 8 – IT-20142 Milan, Italy

**Keywords:** night shift work, breast cancer, DNA methylation, telomere length, female nurses

## Abstract

Increased breast cancer risk has been reported in some night shift (NS) workers but underlying biological mechanisms are still unclear. We assessed the association between NS work and DNA methylation of tumor suppressor (*TP53*, *CDKN2A*, *BRCA1*, *BRCA2*) and estrogen receptor (*ESR1*, *ESR2*) genes, methylation of repetitive elements (*LINE-1*, *Alu*), and telomere length (TL). Forty six female nurses employed in NS for at least two years were matched by age (30–45 years) and length of service (≥1 year) with 51 female colleagues not working in NS. Each subject underwent a semi-structured interview and gave a blood sample. We applied linear regression and spline models adjusted for age, BMI, smoking habit, oral contraceptive use, parity and marital status/age at marriage. Currently working in NS was associated with *ESR1* hypomethylation (β: −1.85 (95%CI: −3.03; −0.67), *p* = 0.003). In current and former NS workers we observed *TP53* (−0.93 (−1.73; −0.12), *p* = 0.03) and *BRCA1* (−1.14 (−1.71; −0.58), *p* <0.001) hypomethylation. We found an increase between TL and number of years in NS in subjects employed in NS <12 years (0.06 (0.03; 0.09), *p* <0.001), while a decrease if employed in NS ≥12 years (−0.07 −0.10; −0.04), *p* <0.001). Our findings show NS-associated markers potentially involved in cellular aging, genomic instability, and cancer development.

## 1. Introduction

Shift work which causes circadian disruption has been classified as “probably carcinogenic to humans” (Group 2A) by the International Agency for Research on Cancer (IARC) [1], on the basis of limited evidence in humans. Most epidemiological studies considered in IARC evaluation examined breast cancer risk, with the most relevant evidence coming from two prospective cohort studies that showed an increased risk in a subgroup of female nurses after over 20–30 years of rotating night shift work [2,3]. 

Several other investigations have been published since, most of which collectively indicate a tendency of increased risk of breast cancer, especially in workers employed in night shifts for several years [4] or in schedules characterized by many consecutive night shifts [5]. Nonetheless, an overall heterogeneity characterizes these studies, mostly due to lack of a standard definition of exposure, differences in study design, missing information on chronotype, differences in the menopausal status of the investigated populations and in breast cancer subtypes [6]. 

The mechanisms hypothesized for the association between circadian disruption and the induction and/or promotion of malignant tumors are multifactorial [7]: repeated phase shifting and consequent defects in circadian cell-cycle regulation may favor uncontrolled cell growth; melatonin suppression may lead to an up-regulation of the effects of estrogen upon the breast epithelial cell; sleep deprivation is known to suppress immune surveillance, thus potentially allowing the formation and/or growth of malignant clones. However, none of the identified factors seems to be the only responsible for the moderately increased cancer rate among shift workers. 

Epigenetic mechanisms mediate the adaptation of the genome to environmental stimuli, and epigenetic alterations can contribute to the development of disease phenotypes resembling genetic mutations [8]. In recent years, a growing body of evidence has focused on epigenetic modifications as potential mechanism underlying several diseases, including cancer, cardiovascular, respiratory, and neurodegenerative diseases [9,10]. DNA methylation, a process characterized by addition of methyl (-CH3) groups to the DNA molecule, represents the most frequent epigenetic modifications in eukaryotic DNA [11] and is involved in regulating many cellular processes, including chromosome stability and gene transcription [12]. Altered DNA methylation patterns in the promoter regions of several genes have been associated with increased cancer risk [13]. In addition, reduced methylation of repetitive elements such as *LINE-1* and *Alu* has been associated with genomic instability [14,15]. On the other hand, telomeres are the non-coding terminal regions of the chromosomes which consist of highly repeated sequences. Their length is influenced by several physiologic, lifestyle, and environmental factors, including ageing, smoking habit, psychological stress, overweight/obesity, and exposure to pollutants [16]. Altered telomere length has been also associated with night shift work [17,18,19]. In addition, genomic instability following telomere shortening represents a recognized mechanism of cancer development [20].

We thus established the present study to assess the association between night shift work and molecular alterations potentially related to a higher carcinogenic risk. We focused our analysis on DNA methylation of estrogen receptor genes (*ESR1*, *ESR2*) and the tumor suppressor genes *TP53*, *CDKN2A*, *BRCA1*, *BRCA2* which play a relevant role in key cellular processes such as cell growth, apoptosis, and DNA repair and have been associated (with different mechanisms) with increased breast cancer risk [21,22,23,24]; methylation of repetitive elements (*LINE-1*, *Alu*); and telomere length.

## 2. Materials and Methods 

### 2.1. Study Population 

The study population has been recruited, on a voluntary basis, among the female nurses employed at the Fondazione IRCCS Ca’ Granda Policlinico Hospital in Milan, Italy. Invitation for participation was proposed by the Occupational Health Physician of the hospital, during the routine visits of the workers’ health surveillance program. All subjects gave their informed consent for inclusion before they participated in the study. The study was conducted in accordance with the Declaration of Helsinki [25], and the protocol was approved by the Institutional Review Board of the Policlinico Hospital.

To be eligible for inclusion in the study, subjects had to be female nurses of Caucasian ethnicity, aged 30–45 years, and with a length of service of at least 1 year. Subjects were excluded if, at the time of the visit, they were affected by cancer, neurological diseases (e.g., multiple sclerosis, Alzheimer’s or Parkinson’s disease, epilepsy), or acute relapses of systemic diseases (e.g., cardiovascular diseases, diabetes). Other exclusion criteria were: antihypertensive or steroid drug assumption, pregnancy or menopausal status, and body mass index (BMI) >30.

Current night shift workers were defined as workers who had been employed in shifts including nights for at least 2 years, and were matched to non-night shift workers by age and length of service.

### 2.2. Collection of Personal Data and Biological Samples

After all subjects gave their consent to participate in the study, they underwent a semi-structured interview, which included a modified version of the Standard Shiftwork Index [26], to collect information on demographic characteristics, health status (current or past diseases, medical drugs, etc.), lifestyle (diet, alcohol consumption, smoking habit, etc.), occurrence of breast cancer among family members, gynecological history (parity, oral contraceptive use, characteristics of the menstrual cycle, etc.), and work (job title, length of service, etc.), with a particular focus on shift work schedule and duration.

Each subject was withdrawn a 12 ml blood sample for subsequent laboratory analyses (see below). Blood collection (in EDTA tubes) occurred at the end of the night shift (between 7:15 and 7:45 in the morning) for night shift workers and at the beginning of the day shift for non-night shift workers. 

### 2.3. DNA Extraction and Methylation Analysis

Genomic DNA was isolated from buffy coat using a commercial kit (Wizard Genomic DNA Purification Kit, Promega, Madison, WI, USA) following manufacturer procedures, and aliquoted at a concentration of 25 ng/µL.

The analysis of DNA methylation was performed following a slightly modified version of a previously described procedure [27]. Briefly, 500 ng of DNA (concentration 25 ng/μL) were treated with EZ DNA Methylation-Gold™ Kit (Zymo Research, Orange, CA, USA) according to the manufacturer’s protocol. Bisulphite-treated DNA was eluted in 300 μl of M-Elution Buffer. PCR reactions (50 μL volume) were carried out with 25 μL of Hot Start GoTaq Green Master mix (Promega), 1 pmol of forward primer, 1 pmol of reverse primer and 25 ng of bisulfite-treated genomic DNA. PCR cycling conditions and primer sequences are provided in Table S1; CpG islands analyzed for gene-specific methylation are reported in Table S2. Biotin-labeled primers (forward or reverse, depending on the assay) were used to purify the final PCR product with Sepharose beads. The PCR product was bound to Streptavidin Sepharose beads (Amersham Biosciences, Uppsala, Sweden), purified, washed, denatured with 0.2 M NaOH, and washed again with the Pyrosequencing Vacuum Prep Tool (Pyrosequencing, Inc., Westborough, MA, USA), according to manufacturer’s instructions. Pyrosequencing primer (0.3 μΜ) was annealed to the purified single-stranded PCR products, and pyrosequencing was performed with the PyroMark MD System (Pyrosequencing, Inc. Westborough, MA, USA). Methylation levels were expressed as the percentage of cytosines that were methylated, determined as the number of methylated cytosines divided by the sum of methylated and unmethylated cytosines, multiplied by 100 (% 5-methyl-Cytosine).

### 2.4. Telomere Length Analysis

Telomere length was measured by Real-time PCR, according to methods described previously [28,29,30]. In brief, the relative telomere length was measured by determining the ratio of telomeric repeat copy number (T) to a nuclear single copy gene (S, human beta-globin gene, *HBB*) copy number (T/S ratio) in a given sample relative to a reference pooled DNA used to generate a standard curve, which was inserted in each PCR run. Primer sequences have been reported elsewhere [28]. The reference pool DNA was prepared from 10 DNA samples (1µg DNA for each sample). A fresh standard curve prepared from the pooled DNA, ranging from 24 ng/µL to 0.1875 ng/µL (serial dilutions 1:2), was included in every “T” and “S” PCR run. For each sample, 22.5 ng of DNA were used as template and each reaction was run tripled. All PCR reactions were performed on a 7900HT Fast Real-Time PCR System (Applied Biosystems, Waltham, MA USA). At the end of each real-time PCR reaction, a melting curve was added in order to confirm the amplification specificity and the absence of primer dimers. The average of the three T measurements was divided by the average of the three S measurements to calculate the T/S ratio for each sample.

### 2.5. Statistical Analysis

We used standard descriptive statistics [means, standard deviations (SDs), and proportions] to summarize data. Differences in variables distribution between current night shift workers vs. non-night shift workers were assessed using Student’s t for continuous variables and chi-squared and Fisher’s exact tests for categorical variables. Association between DNA methylation, telomere length and night shift work [either considered as current vs. non-night shift workers or as ever vs. never night shift workers] was assessed by applying linear regression models both unadjusted and adjusted for age, BMI, smoking habit (current vs. former/never), and oral contraceptive use (yes vs. no). Since we missed information on age at first pregnancy, we created an interaction term between parity and a variable identifying marital status/age at marriage (0 = not married, 1 = married at 30 years of age or older, 2 = married at an age between 25 and 29 years, 3 = married at less than 25 years of age), which we additionally adjusted for in the models. 

To study the association between the total duration of night shift work (in current and former night shift workers) and the molecular outcomes of interest, we flexibly modeled the variable “number of years in night shifts” (NYNS) as a restricted cubic spline with four knots at values 0, 6, 9, and 17 (corresponding to the 10th/25th, 50th, 75th, and 90th percentile of the variable distribution, respectively). We then fitted a regression model including the splines, all the above mentioned adjustment covariates (i.e., age, BMI, smoking habit, oral contraceptive use, and an interaction term between parity and marital status/age at marriage), and a dichotomous variable identifying ever vs. never night shift workers, and plotted the results to inspect data patterns. If a particular pattern was evident, we additionally fit linear spline models (with the same adjustment variables) allowing the slope of the function to change at predefined bending points, to better capture and describe the most prominent features of the exposure-response association.

Results were expressed as regression coefficients (β), which take the unit of measure of the investigated outcome variable (i.e., methylation percentage or telomere length), and corresponding 95% confidence intervals (95% CI). Analyses were performed using STATA (StataCorp. 2017. Stata Statistical Software: Release 15. College Station, TX: StataCorp LLC). 

## 3. Results

We recruited 46 current night shift workers and 51 workers not currently employed in night shifts, whose main characteristics are summarized in Table 1. Our study population had a mean age of 35.9 years (± 5.4) and an average length of service of 11.7 years (± 6.9): these two variables, together with BMI, did not significantly vary between the two groups. Night shift workers smoked and used oral contraceptives in slightly higher proportions, and 89% of them did not have children (*p* = 0.003). 

Twenty-three non-night shift workers had previously worked in night shifts. Among ever night shift workers with available information (N = 66), mean NYNS was 9.5 (± 5.3), with a minimum of 2 and a maximum of 24 years.

Summary statistics for DNA methylation (%) and telomere length (T/S) are reported in Table S3. 

When investigating whether current night shift work could influence DNA methylation or telomere length, the fully adjusted regression models mostly returned null results, except for a clear reduction in the methylation of *ESR1* associated with current night shifts, which was confirmed in both unadjusted (β = −1.67, 95%CI: −2.58 ; −0.76, *p* <0.001) and adjusted (β = −1.85, 95%CI: −3.03 ; −0.67, *p* = 0.003) models (Table 2).

Similar results for *ESR1* associated with night shift work were obtained also when considering both current and former night shift workers, with an adjusted regression coefficient of −1.52 (95%CI: −2.81; −0.22, *p* = 0.02). This analysis also showed a hypomethylation in *TP53* (β = −0.93, 95%CI: −1.73; −0.12, p = 0.03) and *BRCA1* (β = −1.14, 95%CI: −1.71 ; −0.58, *p* <0.001) among ever night shift workers. No other pattern was apparent, except for an increase in the methylation of *ESR2* associated with night shifts that, however, presented very wide confidence intervals and was far from statistical significance (Table 3).

**Figure 1:**
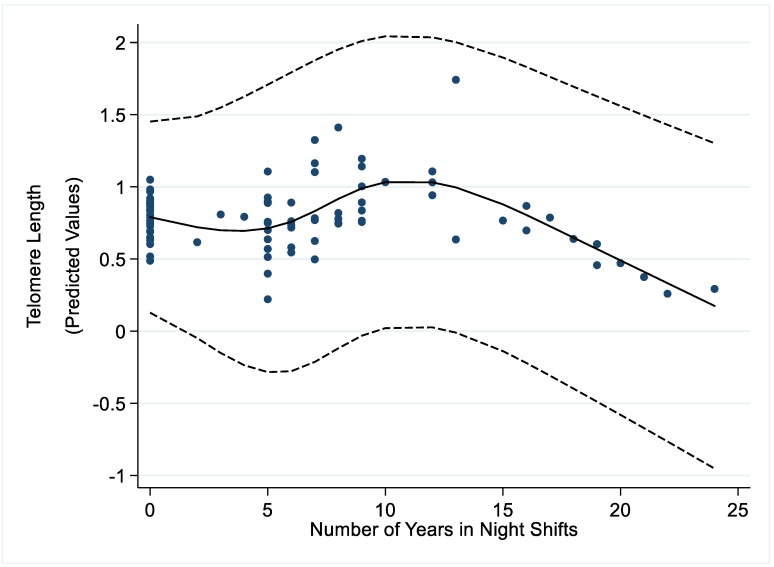
Association^*^ between number of years in night shifts and telomere length.* Number of years in night shifts modeled as a restricted cubic spline with four knots at values 0, 6, 9, 17; other variables in the model include ever/never night shifts, age, BMI, smoking habit, oral contraceptive use, and an interaction term between parity and marital status/age at marriage.

Spline models did not show evidence of an association between NYNS in current and former night shift workers and the investigated epigenetic outcomes, with curves depicting the exposure-response function that were mostly flat (Figures S1–S8). On the other hand, the model inspecting the relationship between NYNS and telomere length did show a pattern of the data, with an increase in telomere length for values of NYNS up to about 12 years, and a subsequent decrease (Figure 1).

We then modeled the variable NYNS as a linear spline allowing the slope of the function to change at 12 years: we observed an increase in telomere length in subjects having worked in night shifts for less than 12 years (β = 0.06, 95%CI: 0.03 ; 0.09, *p* <0.001) and a decrease in nurses employed in night shifts for 12 years or more (β = −0.07, 95%CI; −0.10 ; −0.04, *p* <0.001). No association was observed between linear splines of NYNS and the other investigated epigenetic outcomes (Table S4). A sensitivity analysis restricted to ever night shift workers returned similar results (not shown).

## 4. Discussion

In a study including almost 100 female nurses, we documented an association between night shift work and epigenetic and molecular alterations potentially related to a higher carcinogenic risk.

In particular, we observed a reduced methylation of *ESR1* associated with night shift work, both when analyzing current night shift workers vs. non-night shift workers, and when comparing ever vs. never night shift workers. A recent study on breast cancer cells suggested that low levels of *ESR1* promoters methylation may favor *ESR1* gene expression (mediated by binding of progesterone to the promoter region) [31]. *ESR1* encodes estrogen-receptor [ER] alpha, whose stimulation by estrogens is known to be associated with an increased proliferation of breast tissue [32]. Our results might thus indicate an increased breast tissue sensitivity to estrogenic stimulation, which is a well-known risk factor for breast cancer [33]. A recent cohort study on over 400 Chilean girls found methylation of the ER-Alpha gene to be inversely associated with some indicators of breast composition, including total breast volume, fibroglandular volume, and percent fibroglandular volume. Since increased proportion of dense breast tissue in adults represents a strong risk factor for breast cancer [34], the authors suggest that the methylation profile of *ESR1* may modulate adolescent response to estrogen and may thus influence breast cancer risk in adulthood [35]. Although our population is older, this evidence might contribute to interpret our results in the light of the complex relationship between estrogen sensitivity and increased cancer risk. In addition, our results on *ESR1* might also represent a signal of altered melatonin secretion, which has been extensively documented in night shift workers [36], as melatonin is considered to play a role in the regulation of the ER expression [37].

When including in our analyses current and former night shift workers, we observed a reduced methylation of *TP53* and *BRCA1* genes, both encoding tumor suppressors that prevent the transformation of normal cells to cancer cells: p53 (encoded by *TP53* gene) regulates cell-cycle progression by means of several mechanisms, such as apoptosis, senescence, DNA repair and differentiation [38]; *BRCA1* is a well-known breast cancer susceptibility gene, and its encoded protein (together with that of *BRCA2*) is involved in the repair of chromosomal damage, displaying its functions in the error-free repair of DNA double-strand breaks [39]. Although not directly related to breast cancer, some evidences have been suggesting that hypomethylation of *BRCA1* might be associated with an increased carcinogenic risk. In particular, a case-control study conducted in France found significantly decreased *BRCA1* methylation in peripheral blood cells of 51 sporadic ovarian cancer cases compared with 349 healthy female controls [40]. Given all the above, we could speculate that reduction of *TP53* and *BRCA1* methylation might be induced to counterbalance defects in circadian cell-cycle regulation which are possibly involved in uncontrolled cell growth, in night shift workers. These findings might be part of a bigger picture where long-term night shift work has been associated with diverse epigenetic alterations [41].

We observed a reduced telomere length with increasing NYNS in nurses with at least 12 years of night shifts. Genomic instability following telomere shortening is a well-established mechanism of tumor development [20] and some studies documented an increased cancer risk among subjects with reduced telomere length [42]. Nonetheless, available data on the association between telomere length and breast cancer still remain inconsistent [43,44,45]. On the other hand, findings correlating telomere shortening with breast cancer stage and progression seem more coherent [46,47,48]. In particular, the finding on telomere shortening in subjects with longer duration of total night shift work is concordant with a recent case-control study conducted on more than 560 breast cancer cases and about 600 controls, which showed a reduced telomere length in female nurses employed in (consecutive) night shifts for at least 5 years [49].

We did not find any significant association between night shift work and methylation of repetitive elements in adjusted models; these null findings are somewhat similar to what observed in another investigation conducted by our research team on a group of 150 workers, including 100 shift workers, which did not show any evidence of a relationship between shift work and methylation of either *LINE-1* or *Alu* [50]. On the other hand, a recent study conducted on a tissue-isolated xenograft model of human prostate cancer showed that *LINE-1* expression was suppressed by melatonin [51], and that the overexpression of melatonin receptor 1 suppressed *LINE-1* and *Alu* mobilization (driven by *LINE-1*) in cancer cells; the authors hypothesized that loss of *LINE-1* regulation following light at night exposure (and subsequent suppression of melatonin production) might represent an underlying mechanism for the higher cancer incidence rates in people experiencing light at night, such as shift workers [52]. However, evidences produced on this specific topic are still scarce and preclude firm conclusions. 

Our work has several strengths. First of all, it looks at various molecular outcomes in the attempt to offer some insights in the understanding of the mechanisms that might underlie the association between night shift work and breast cancer. As we used flexible models to evaluate the relationship between molecular markers and a surrogate of cumulative exposure such as NYNS, we did not constrain our data to *a priori* assumptions on the linearity of the investigated associations [53]. Still, the use of linear splines allowed us to describe more easily the most prominent features of the association between NYNS and the analyzed outcomes, telomere length in particular. In addition, we were able to adjust for several individual confounders, including parity and a proxy for age at first pregnancy, which are both known to influence breast cancer risk [54].

Our study has also limitations. The small number of the recruited subjects hampers our findings with some uncertainty and precludes sound conclusions. Secondly, all information regarding the main exposure of interest (i.e. characteristics of the shifts) as well as all the confounders were self-reported, even if the absence of a pathologic outcome should bring to avoid major distortions. Thirdly, the characterization of the exposure lacked some important pieces of information (such as number of subsequent night shifts, precise scheme of the shift schedule, etc.). Lastly, the changes we observed are small in size; nonetheless, apparently small methylation differences (when expressed as percentage over the total number of cytosine in the considered position) involve indeed many blood cells and a large number of DNA molecules whose expression might be altered.

## 5. Conclusions

Our findings, although preliminary and based on a small sample, suggest an association between prolonged exposure to night shifts and molecular alterations that might be involved in processes such as cellular aging, genomic instability, and cancer development. Further studies on larger homogenous working populations are warranted (if possible with collection of biological samples), together with a better exposure assessment, taking into account not only the overall duration of night shift work (i.e., the cumulative exposure), but also its intensity, its schedule (including data on resting periods) and, when feasible, information regarding the chronotype of the study population.

## Figures and Tables

**Table 1:** Characteristics of the study population.

Characteristic		Current night shift		
*Mean ± SD, N (%)*	**Total**	**No (51)**	**Yes (46)**	p *
Age	35.9 ± 5.4	36.5 ± 5.3	35.3 ± 5.6	0.31
Length of service	11.8 ± 6.9	12.7 ± 7.3	10.7 ± 6.3	0.17
BMI	22.7 ± 3.2	22.2 ± 3.3	23.2 ± 3.0	0.14
Smoking habit				
Former/Never	67 (71.3)	38 (76.0)	29 (65.9)	
Current	27 (28.7)	12 (24.0)	15 (34.1)	0.36
Oral contraceptive use				
No	58 (63.0)	33 (67.4)	25 (58.1)	
Yes	34 (37.0)	16 (32.6)	18 (41.9)	0.36
Number of children				
0	70 (72.1)	29 (56.9)	41 (89.1)	
1	12 (12.4)	10 (19.6)	2 (4.4)	
2+	15 (15.5)	12 (23.5)	3 (6.5)	0.002
Marital status/Age at marriage				
Not married	55 (56.7)	24 (47.1)	31 (67.4)	
Married at 30+ years	16 (16.5)	11 (21.6)	5 (10.9)	
Married at 25–29 years	20 (20.6)	12 (23.5)	8 (17.4)	
Married at <25 years	6 (6.2)	4 (7.8)	2 (4.3)	0.23

SD: standard deviation. * Student’s t test for continuous variables, chi-squared and Fisher’s exact tests for categorical variables.

**Table 2:** Association between night shift work [current (*N* = 46) vs. non-night shift workers (*N* = 51)] and gene-specific methylation, methylation of repetitive elements, and telomere length.

Biological Markers	Unadjusted			Adjusted *		
	β	(95%CI)	*p*	β	(95%CI)	*p*
***TP53***	−0.32	(−0.93 ; 0.30)	*0.31*	−0.19	(−0.97 ; 0.59)	*0.63*
***CDKN2A***	0.25	(−0.12 ; 0.62)	*0.19*	0.16	(−0.26 ; 0.58)	*0.46*
***BRCA1***	−0.68	(−1.13 ; −0.23)	*0.004*	−0.42	(−1.00 ; 0.15)	*0.15*
***BRCA2***	−0.07	(−0.92 ; 0.78)	*0.87*	−0.15	(−1.26 ; 0.95)	*0.78*
***ESR1***	−1.67	(−2.58 ; −0.76)	*<0.001*	−1.85	(−3.03 ; −0.67)	*0.003*
***ESR2***	−0.12	(−3.13 ; 2.88)	*0.94*	0.47	(−3.24 ; 4.18)	*0.80*
***LINE-1***	−0.17	(−0.58 ; 0.24)	*0.40*	−0.16	(−0.66 ; 0.34)	*0.52*
***Alu***	0.30	(−0.25 ; 0.85)	*0.29*	−0.12	(−0.73 ; 0.50)	*0.71*
**TL**	0.03	(−0.07 ; 0.14)	*0.53*	0.05	(−0.07 ; 0.18)	*0.39*

TL: telomere length. * Linear regression models adjusted for age, BMI, smoking habit, oral contraceptive use, and an interaction term between parity and marital status/age at marriage.

**Table 3:** Association between night shift work (ever (*N* = 69) vs. never (*N* = 28) night shift workers) and gene-specific methylation, methylation of repetitive elements, and telomere length.

Biological Markers	Unadjusted			Adjusted *		
	β	(95%CI)	*p*	β	(95%CI)	*p*
***TP53***	−0.85	(−1.50 ; −0.20)	*0.01*	−0.93	(−1.73 ; −0.12)	*0.03*
***CDKN2A***	0.22	(−0.18 ; 0.63)	*0.27*	0.09	(−0.37 ; 0.54)	*0.71*
***BRCA1***	−1.25	(−1.70 ; −0.80)	*<0.001*	−1.14	(−1.71 ; −0.58)	*<0.001*
***BRCA2***	−0.09	(−1.01 ; 0.83)	*0.85*	−0.31	(−1.47 ; 0.85)	*0.60*
***ESR1***	−1.53	(−2.55 ; −0.51)	*0.004*	−1.52	(−2.81 ; −0.22)	*0.02*
***ESR2***	1.63	(−1.59 ; 4.85)	*0.32*	2.52	(−1.38 ; 6.43)	*0.20*
***LINE-1***	−0.58	(−1.02 ; −0.15)	*0.009*	−0.42	(−0.96 ; 0.11)	*0.12*
***Alu***	−0.001	(−0.61 ; 0.61)	*0.99*	−0.13	(−0.79 ; 0.53)	*0.70*
**TL**	0.02	(−0.10 ; 0.14)	*0.70*	0.03	(−0.10 ; 0.17)	*0.64*

TL: telomere length. * Linear regression models adjusted for age, BMI, smoking habit, oral contraceptive use, and an interaction term between parity and marital status/age at marriage.

## Supplementary Materials

The following are available online at https://www.mdpi.com/1660-4601/16/13/2292/s1, Figure S1: Association between number of years in night shifts and *TP53* methylation, Figure S2: Association between number of years in night shifts and *CDKN2A* methylation, Figure S3: Association between number of years in night shifts and *BRCA1* methylation, Figure S4: Association between number of years in night shifts and *BRCA2* methylation, Figure S5: Association between number of years in night shifts and *ESR1* methylation, Figure S6: Association between number of years in night shifts and *ESR2* methylation, Figure S7: Association between number of years in night shifts and *LINE-1* methylation, Figure S8: Association between number of years in night shifts and *Alu* methylation, Table S1: Primer sequences and polymerase chain reaction conditions for DNA methylation analysis, Table S2: Analyzed CpG islands on gene promoters, Table S3: Summary statistics for methylation (%) of specific genes and repetitive elements, and telomere length (T/S), Table S4: Association between number of years in night shifts and gene-specific methylation, methylation of repetitive elements, and telomere length, according to duration of work in night shifts (< 12 vs. ≥ 12 years).
