# Supplementary material for: Night Shift Work, DNA Methylation and Telomere Length: An Investigation on Hospital Female Nurses
Source: Int J Environ Res Public Health. 2019 Jun 28;16(13):2292. doi: 10.3390/ijerph16132292 (PMC6651131; doi:10.3390/ijerph16132292)
Supplement: Supplementary file 1 [file ijerph-16-02292-s001.pdf]

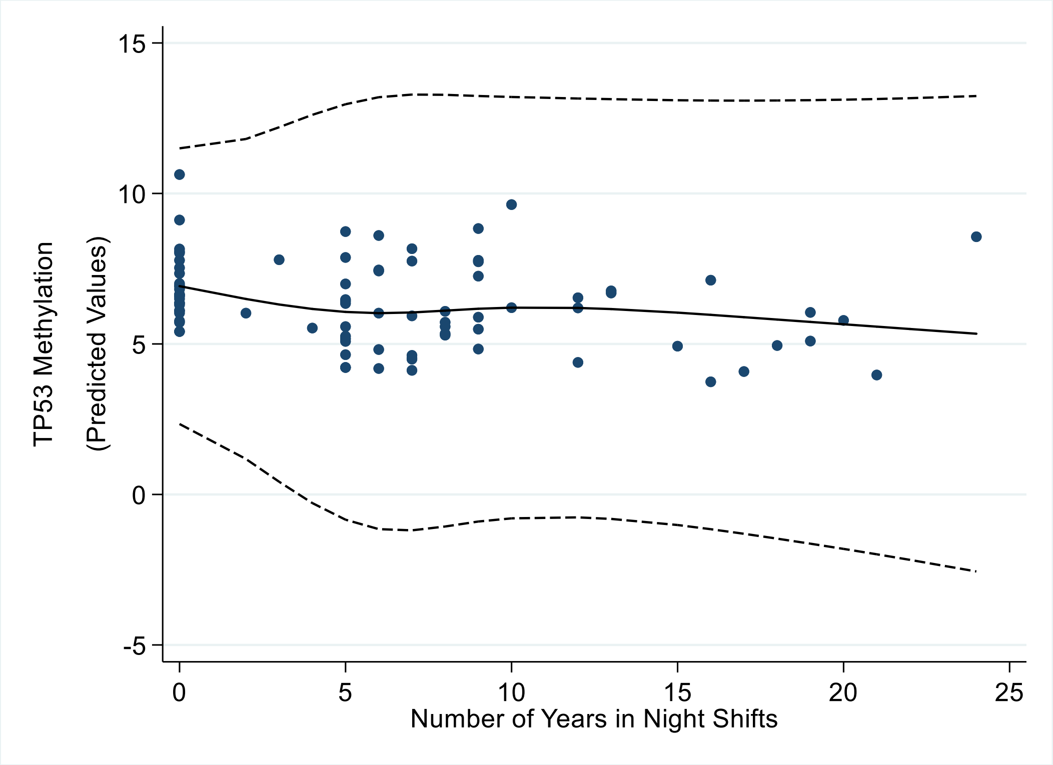

Figure S1. Association* between number of years in night shifts and *TP53* methylation.

* Number of years in night shifts modeled as a restricted cubic spline with four knots at values 0, 6, 9, 17; other variables in the model include ever/never night shift, age, BMI, smoking habit, oral contraceptive use, and an interaction term between parity and marital status/age at marriage.

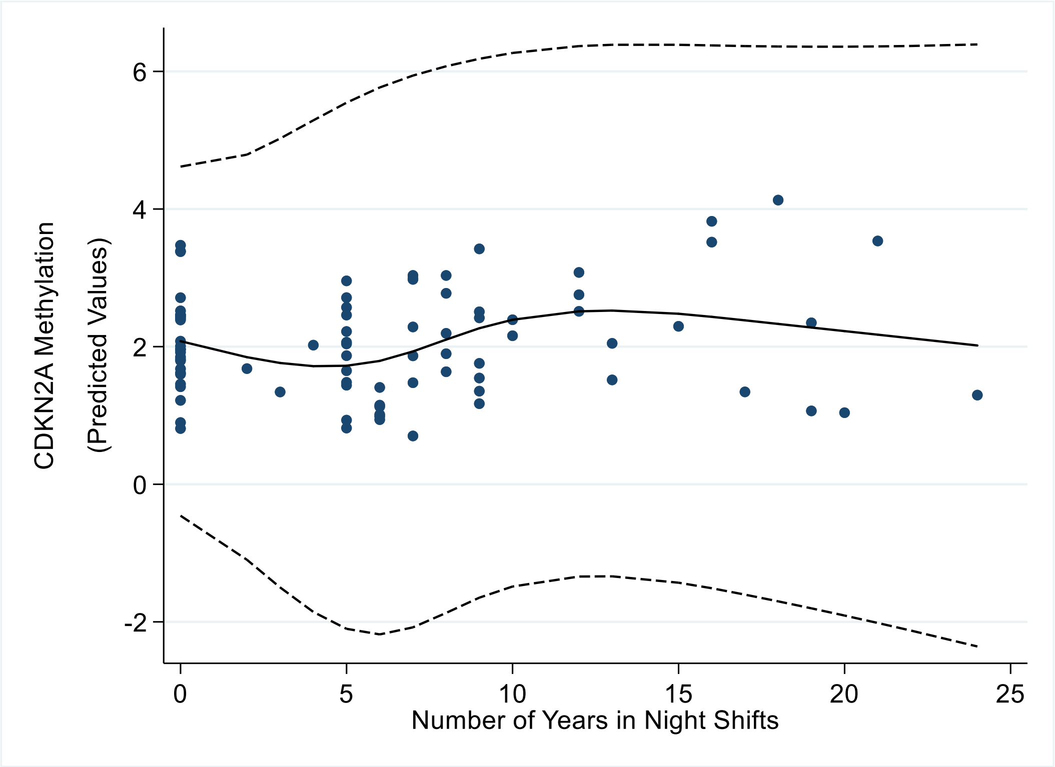

Figure S2. Association* between number of years in night shifts and *CDKN2A* methylation.

* Number of years in night shifts modeled as a restricted cubic spline with four knots at values 0, 6, 9, 17; other variables in the model include ever/never night shift, age, BMI, smoking habit, oral contraceptive use, and an interaction term between parity and marital status/age at marriage.

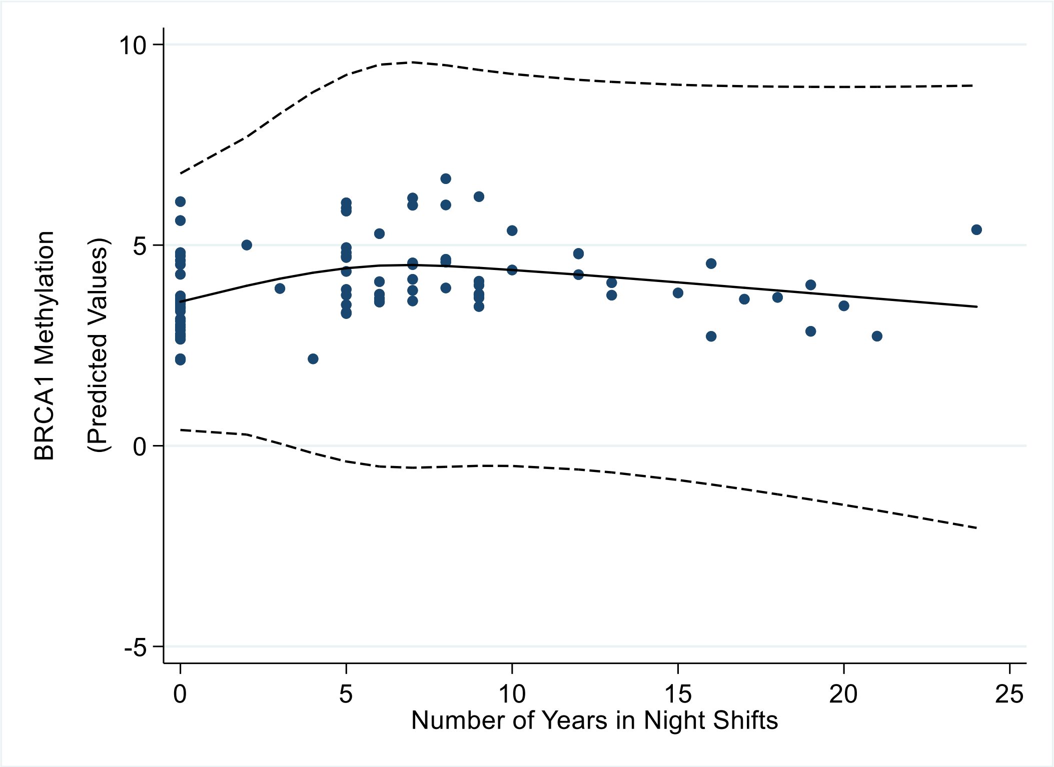

Figure S3. Association* between number of years in night shifts and *BRCA1* methylation.

* Number of years in night shifts modeled as a restricted cubic spline with four knots at values 0, 6, 9, 17; other variables in the model include ever/never night shift, age, BMI, smoking habit, oral contraceptive use, and an interaction term between parity and marital status/age at marriage.

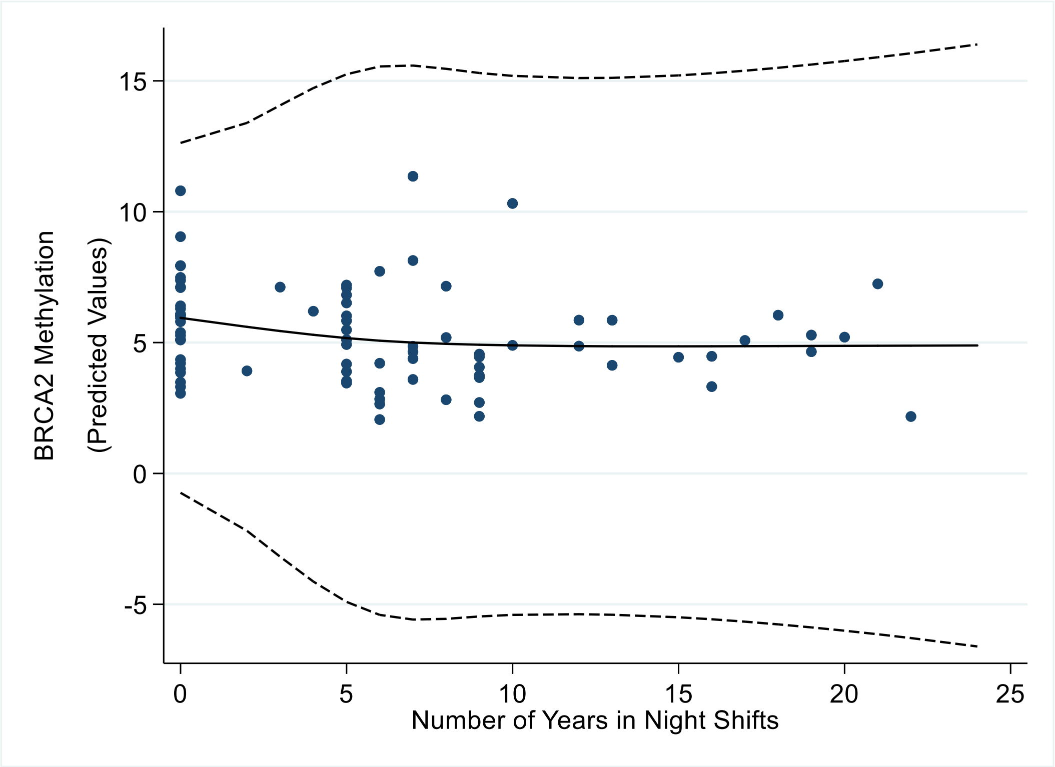

Figure S4. Association* between number of years in night shifts and *BRCA2* methylation.

* Number of years in night shifts modeled as a restricted cubic spline with four knots at values 0, 6, 9, 17; other variables in the model include ever/never night shift, age, BMI, smoking habit, oral contraceptive use, and an interaction term between parity and marital status/age at marriage.

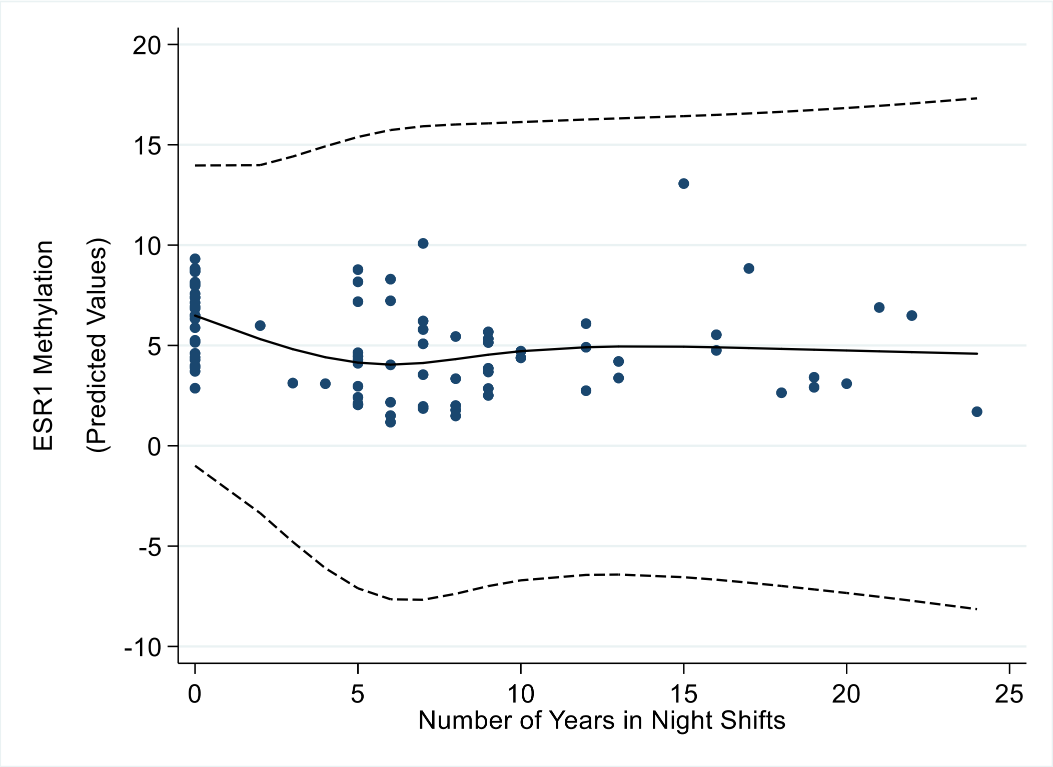

Figure S5. Association* between number of years in night shifts and *ESR1* methylation.

* Number of years in night shifts modeled as a restricted cubic spline with four knots at values 0, 6, 9, 17; other variables in the model include ever/never night shift, age, BMI, smoking habit, oral contraceptive use, and an interaction term between parity and marital status/age at marriage.

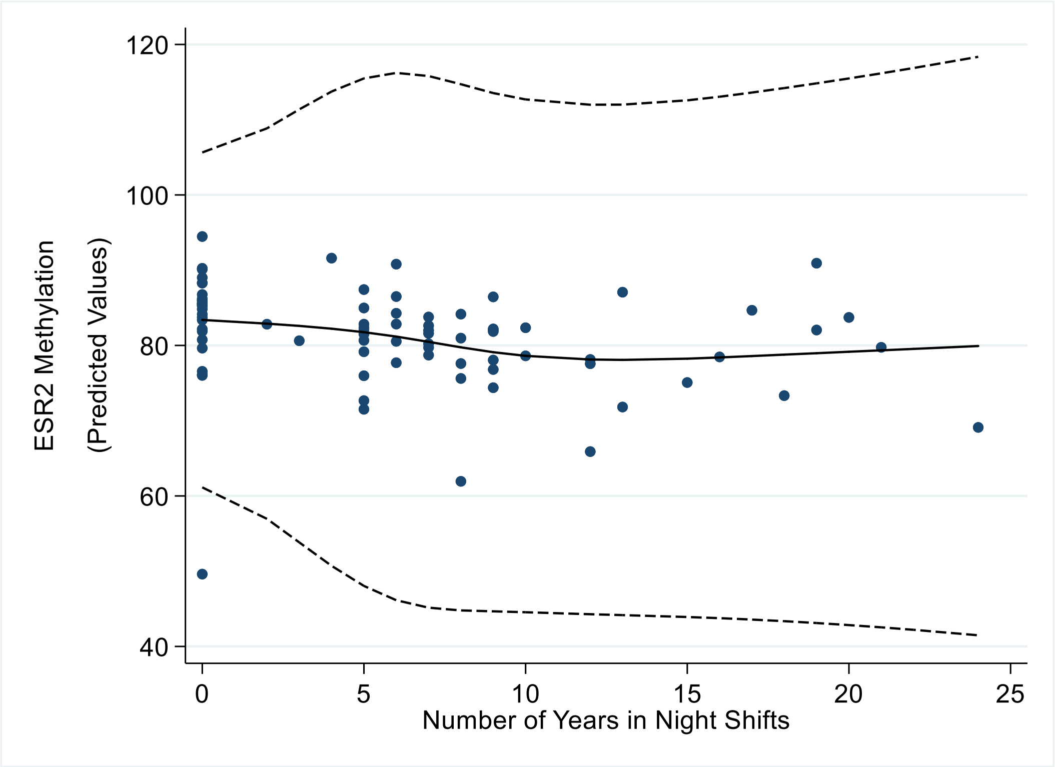

Figure S6. Association* between number of years in night shifts and *ESR2* methylation.

* Number of years in night shifts modeled as a restricted cubic spline with four knots at values 0, 6, 9, 17; other variables in the model include ever/never night shift, age, BMI, smoking habit, oral contraceptive use, and an interaction term between parity and marital status/age at marriage.

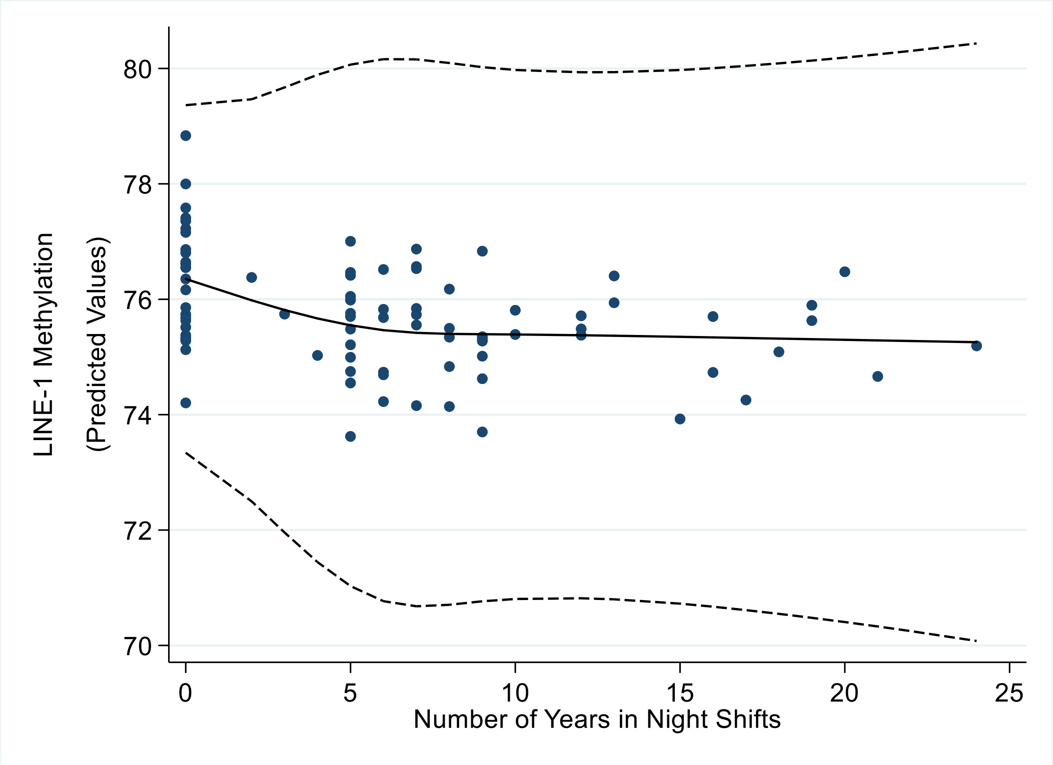

Figure S7. Association* between number of years in night shifts and *LINE-1* methylation.

* Number of years in night shifts modeled as a restricted cubic spline with four knots at values 0, 6, 9, 17; other variables in the model include ever/never night shift, age, BMI, smoking habit, oral contraceptive use, and an interaction term between parity and marital status/age at marriage.

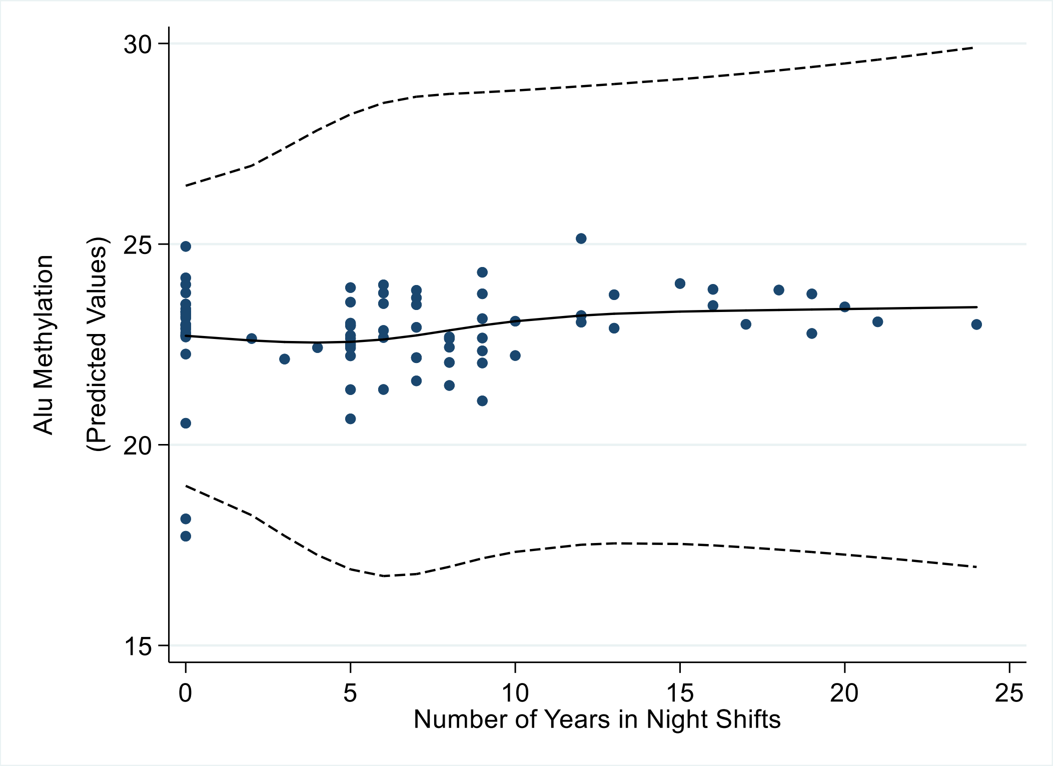

Figure S8. Association* between number of years in night shifts and *Alu* methylation.

* Number of years in night shifts modeled as a restricted cubic spline with four knots at values 0, 6, 9, 17; other variables in the model include ever/never night shift, age, BMI, smoking habit, oral contraceptive use, and an interaction term between parity and marital status/age at marriage.

Table S1. Primer sequences and polymerase chain reaction (PCR) conditions for DNA methylation analysis.

Genes	Forward primer (5′ to 3′)	Reverse primer (5′ to 3′)	Sequencing primer (5′ to 3′)	PCR conditions
TP53	Biotin-TTAGGAGTTTATTTAATTTAGGGAAG	TATCCAACTTTATACCAAAAACCTC	TCCAAAAAACAAATAACTACTAAACTC	95°C for 60s, 57°C for 60s, 72°C for 60s
CDKN2A	AGGGGTTGGTTGGTTATTAG	BIO- CTACCTACTCTCCCCCTCTC	GGTTGGTTATTAGAGGGT	95°C for 30s, 58°C for 40s, 72°C for 30s
BRCA1	ATTTAGAGTAGAGGGTGAAGG	Biotin-TCTATCCCTCCCATCCTCTAATT	TTGAGAAATTTTATAGTTTGTTTT	95°C for 30s, 54°C for 45s, 72°C for 45s
BRCA2	GTTGGGATGTTTGATAAGGAATTT	Biotin- ATCACAAATCTATCCCCTCAC	GTTATATTGAGAAATATT	95°C for 30s, 55°C for 30s, 72°C for 30s
ESR1	GTAGTTTAAGATTTTTTTGGAG	Biotin-CCAAATAATAAAACACCTACTAACC	ATTTGGATAGTAGTAAGTT	95°C for 30s, 48°C for 30s, 72°C for 30s
ESR2	GTGTTGTGGTTAATATTTGGGTAT	Biotin-CACCAAAAACTCTTTTAAAATTCC	TTTTTTTTTTAGTGGTTTAT	95°C for 30s, 60°C for 30s, 72°C for 30s
LINE-1	TTTTGAGTTAGGTGTGGGATATA	Biotin-AAAATCAAAAAATTCCCTTTC	AGTTAGGTGTGGGATATAGT	95°C for 30s, 50°C for 30s, 72°C for 30s

Alu	Biotin-TTTTTATTAAAAATATAAAAATT	CCCAAACTAAAATACAATAA	AATAACTAAAATTACAAAC	96°C for 90s,<br>43°C for 60s,<br>72°C for 120s

Forty-five cycles were performed for all PCR protocols except for the *Alu* one (40 cycles).

Table S2. Analyzed CpG islands on gene promoters.

Genes	Chromosome	Amplicon start	Amplicon end	CpGs (position)	Assembly
TP53	17	7531409	7531628	7531486 7531473 7531469 7531458	hg17
CDKN2A	9	21965321	21965395	21965350<br>21965355<br>21965357<br>21965361<br>21965365<br>21965368<br>21965374	hg19
BRCA1	17	41277339	41277523	41277364<br>41277381<br>41277389<br>41277392<br>41277394	hg19
BRCA2	13	32889486	32889749	32889561<br>32889570<br>32889579<br>32889584<br>32889586<br>32889591<br>32889594<br>32889599	hg19
ESR1	6	152129135	152129444	152129194<br>152129197<br>152129212<br>152129219<br>152129221	hg19
ESR2	14	64749381	64749472	64749426<br>64749448	hg19

Table S3. Summary statistics for methylation (%) of specific genes and repetitive elements, and telomere length (T/S).

Biological Markers	Number of Observations	Mean	Standard Deviation	Minimum	Maximum
TP53	94	6.28	1.49	3.28	10.89
CDKN2A	94	2.04	0.90	0.84	5.11
BRCA1	94	4.08	1.15	2.28	7.69
BRCA2	89	5.26	2.00	2.35	11.91
ESR1	95	5.04	2.38	1.40	13.00
ESR2	91	81.23	7.14	46.87	91.95
LINE-1	93	75.71	0.99	73.34	78.47
Alu	93	22.83	1.34	15.58	24.73
Telomere length	95	0.79	0.26	0.05	1.90

Table S4. Association between number of years in night shifts and gene-specific methylation, methylation of repetitive elements, and telomere length, according to duration of work in night shifts (< 12 vs. ≥ 12 years).

**Biological Markers**	**< 12 years in night shifts**			**≥ 12 years in night shifts**		
	**β***	**(95%CI)**	***p***	**β***	**(95%CI)**	***p***
TP53	0.03	(-0.20 ; 0.25)	*0.82*	-0.07	(-0.27 ; 0.13)	*0.51*
CDKN2A	0.12	(-0.004 ; 0.24)	*0.06*	-0.03	(-0.14 ; 0.09)	*0.63*
BRCA1	0.01	(-0.14 ; 0.17)	*0.88*	-0.08	(-0.23 ; 0.06)	*0.24*
BRCA2	-0.05	(-0.40 ; 0.30)	*0.77*	-0.004	(-0.32 ; 0.31)	*0.98*
ESR1	0.06	(-0.31 ; 0.42)	*0.75*	0.02	(-0.29 ; 0.33)	*0.90*
ESR2	-0.66	(-1.74 ; 0.42)	*0.23*	0.17	(-0.81 ; 1.14)	*0.73*
LINE-1	-0.04	(-0.19 ; 0.11)	*0.58*	-0.003	(-0.14 ; 0.13)	*0.97*
Alu	0.10	(-0.08 ; 0.28)	*0.29*	0.02	(-0.14 ; 0.19)	*0.79*
**TL**	0.06	(0.03 ; 0.09)	*< 0.001*	-0.07	(-0.10 ; -0.04)	*< 0.001*

*TL: telomere length. * Number of years in night shifts modeled as linear spline allowing the slope of the function to change at 12 years; other variables in the model include ever/never night shift, age, BMI, smoking habit, oral contraceptive use, and an interaction term between parity and marital status/age at marriage.*
